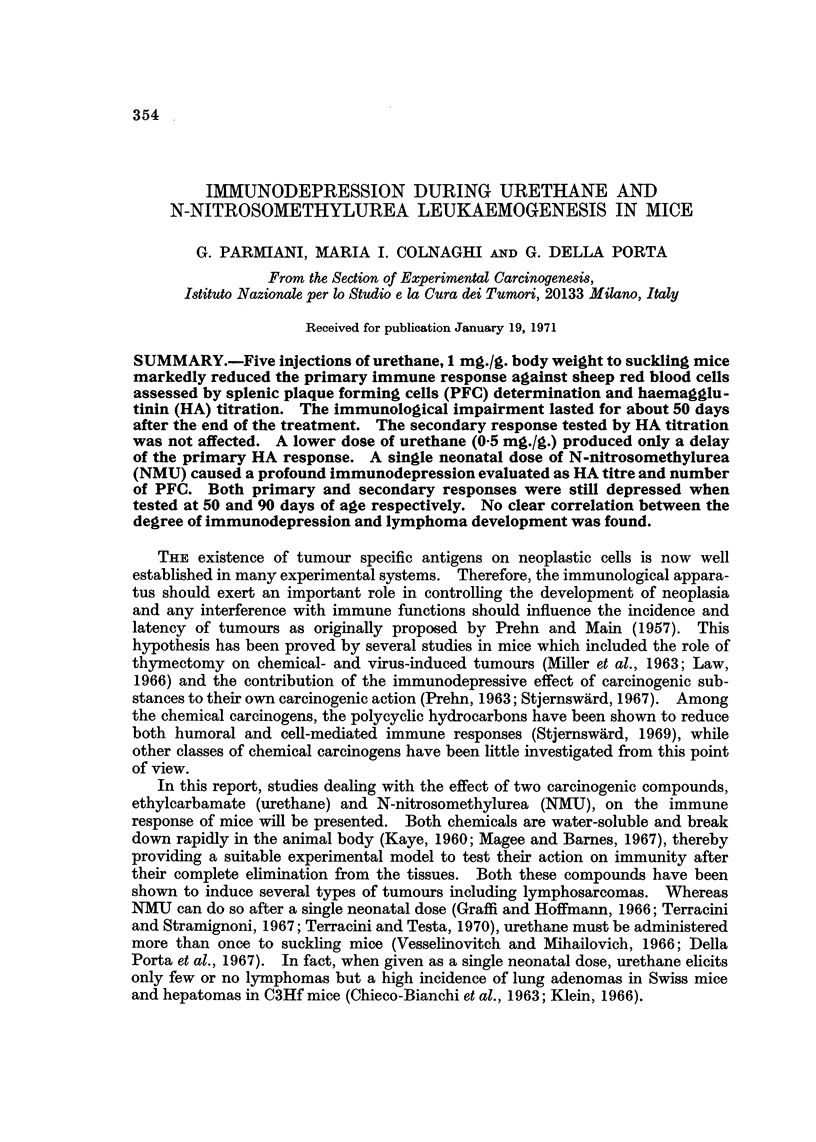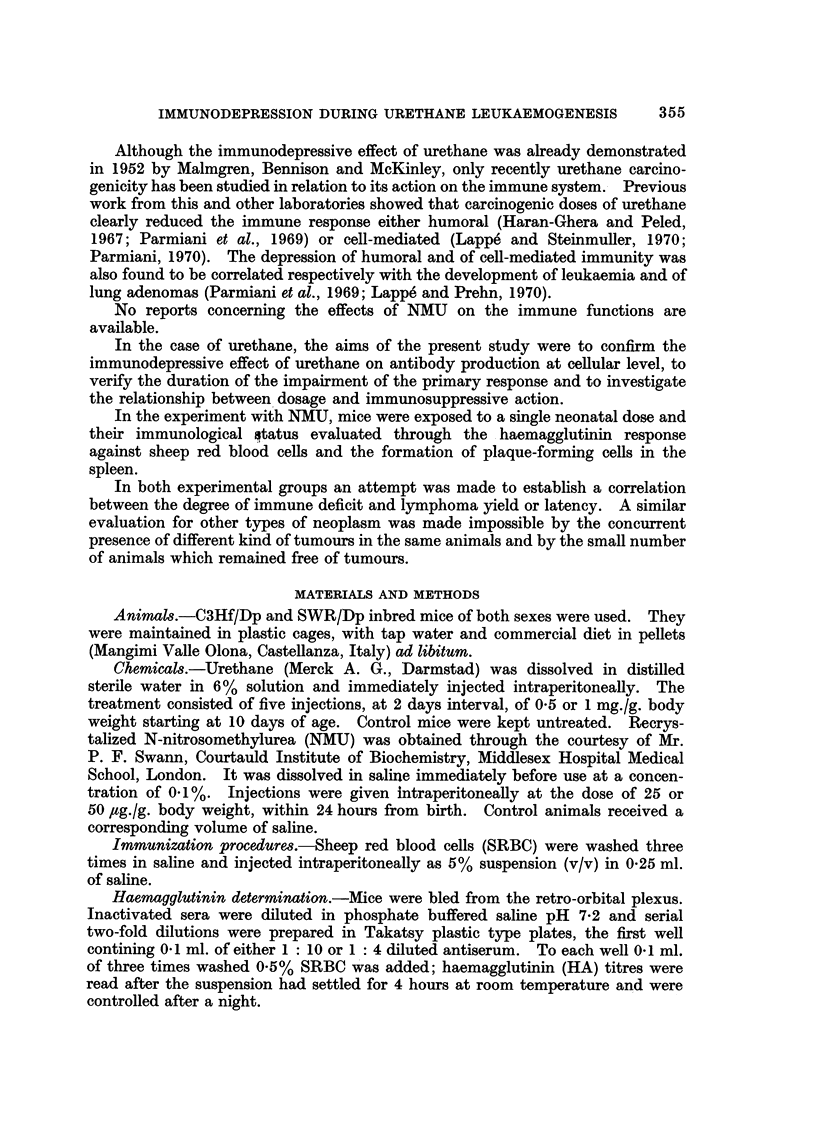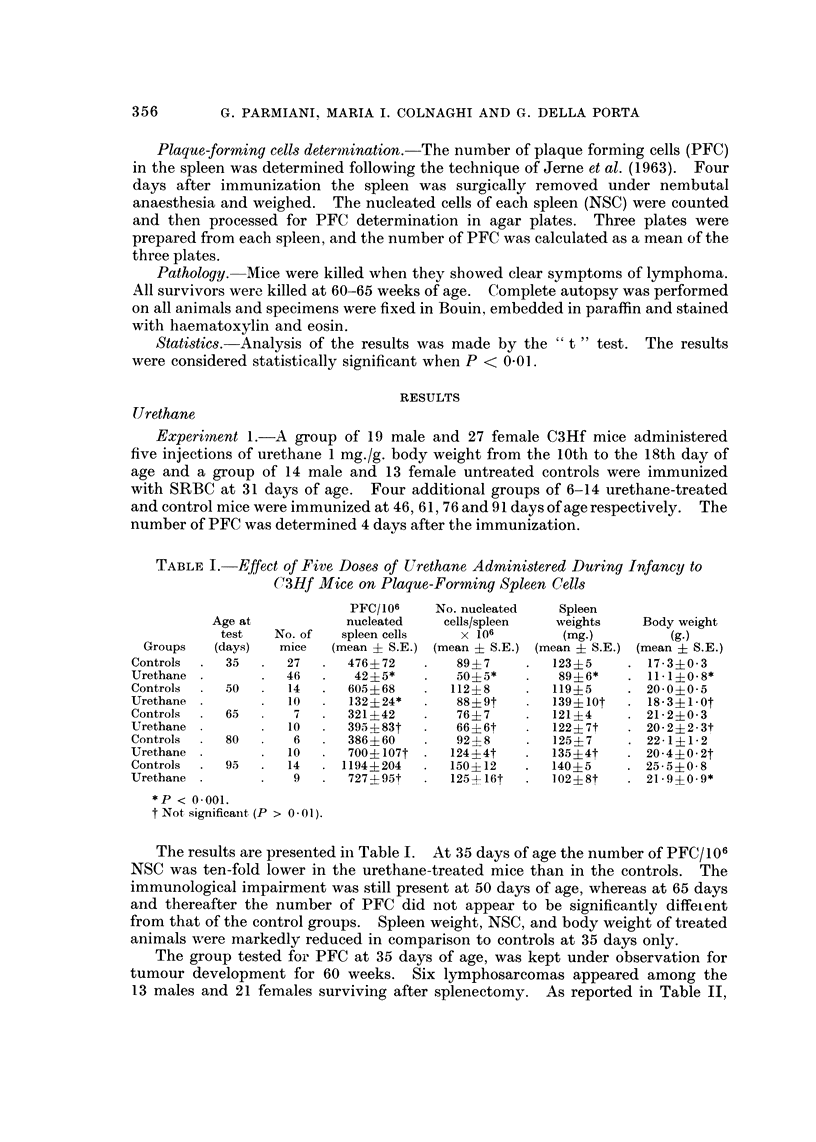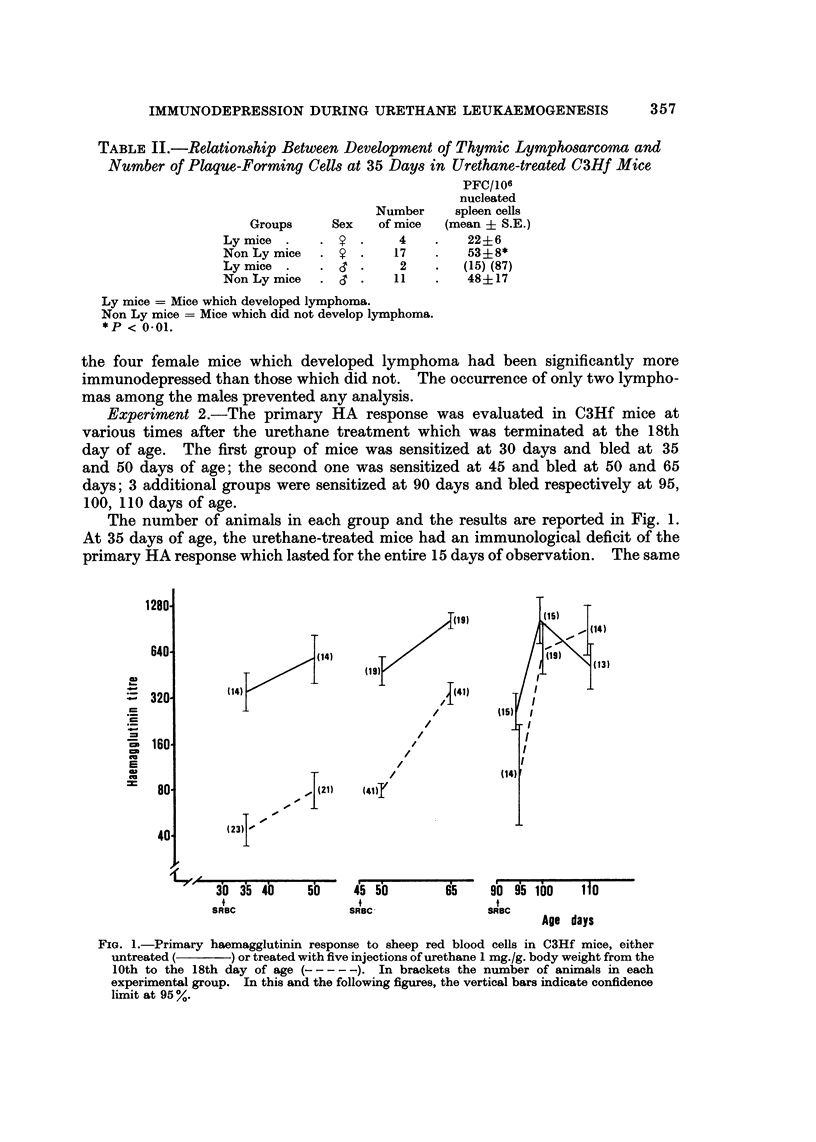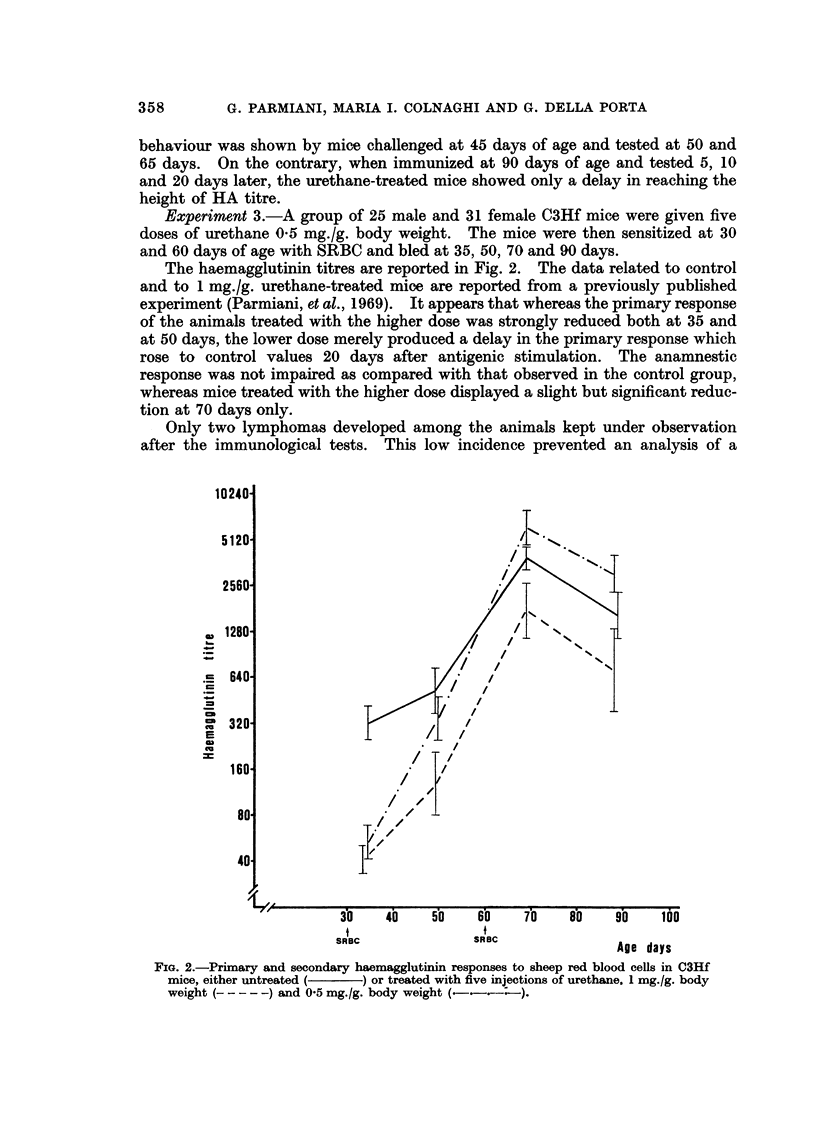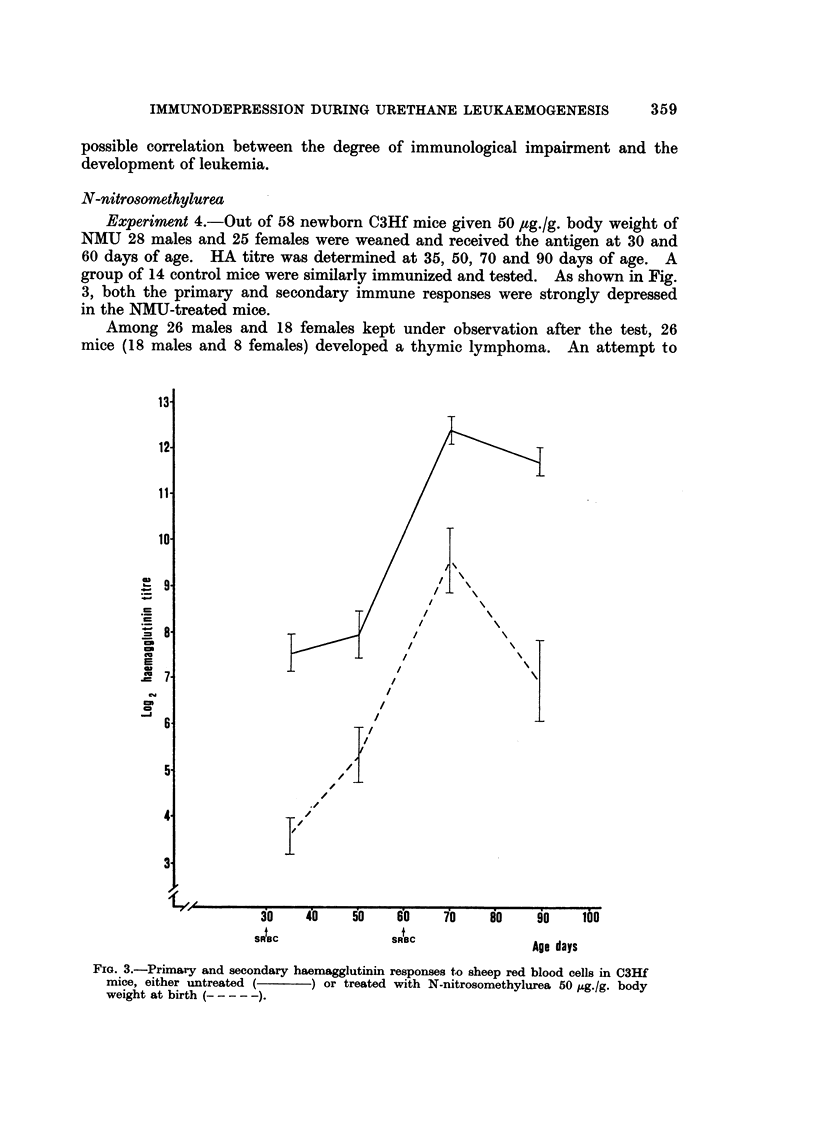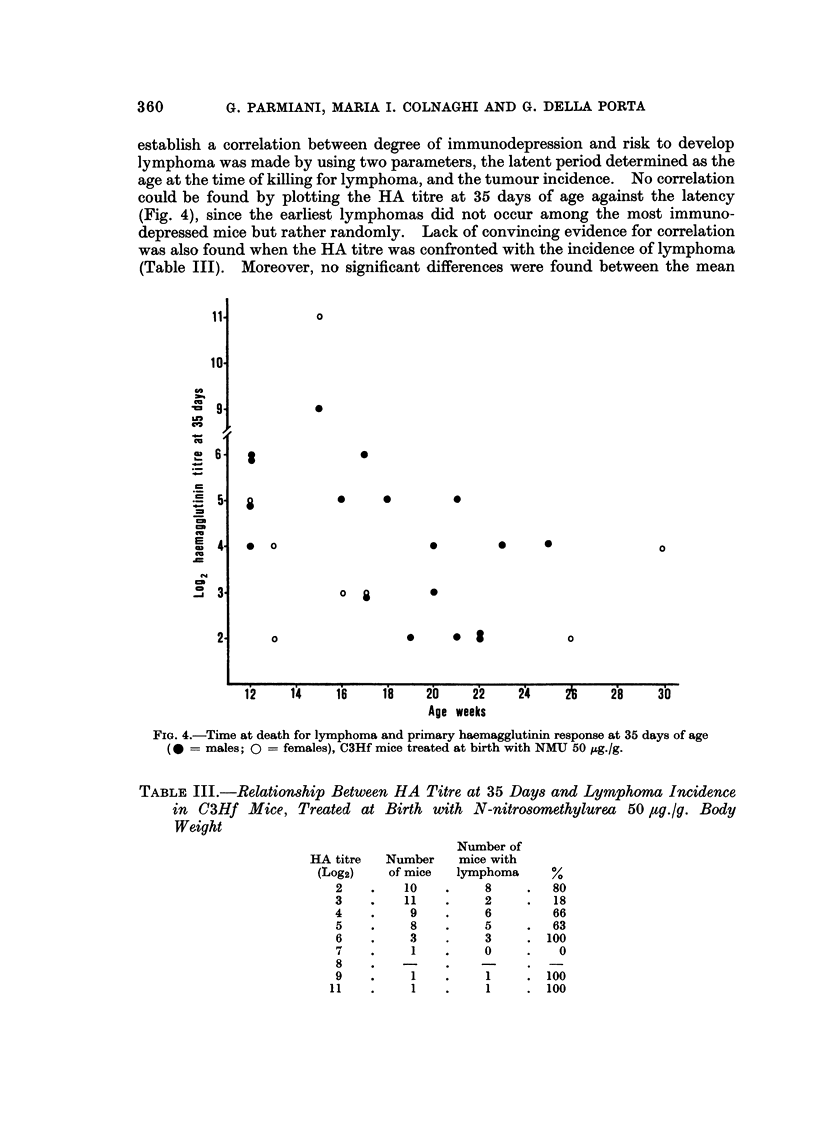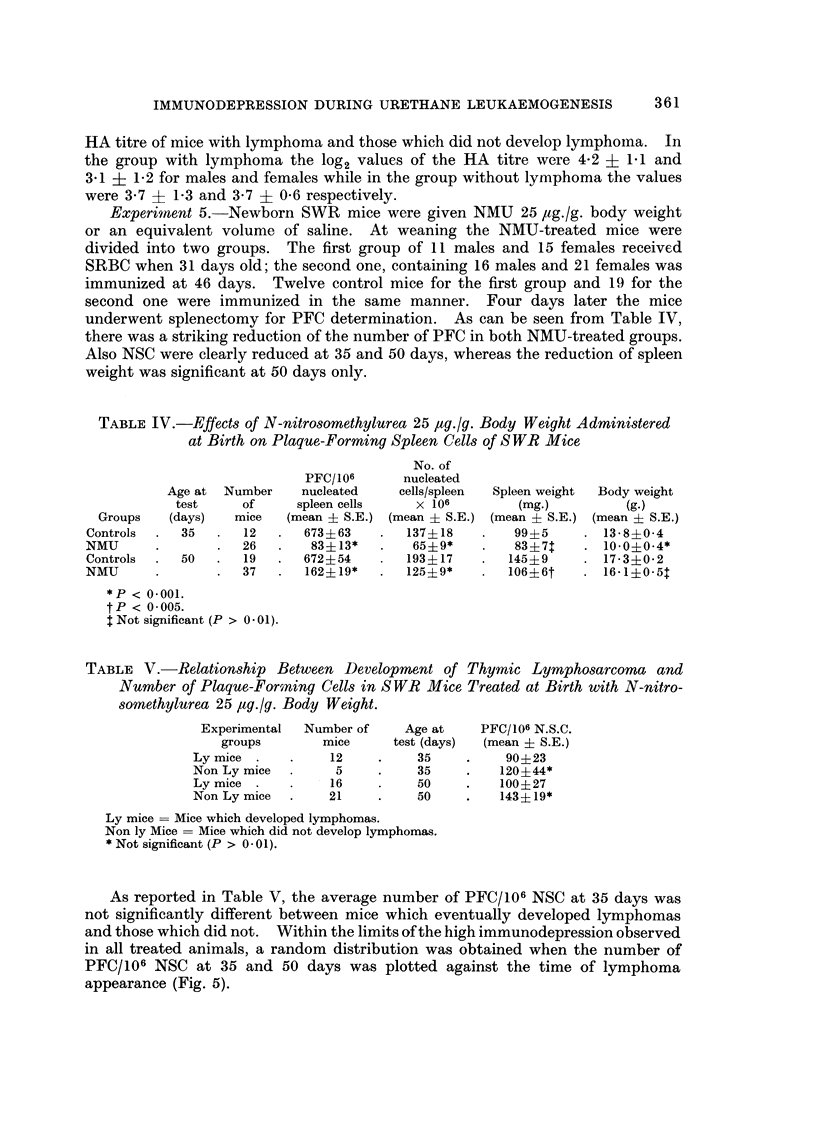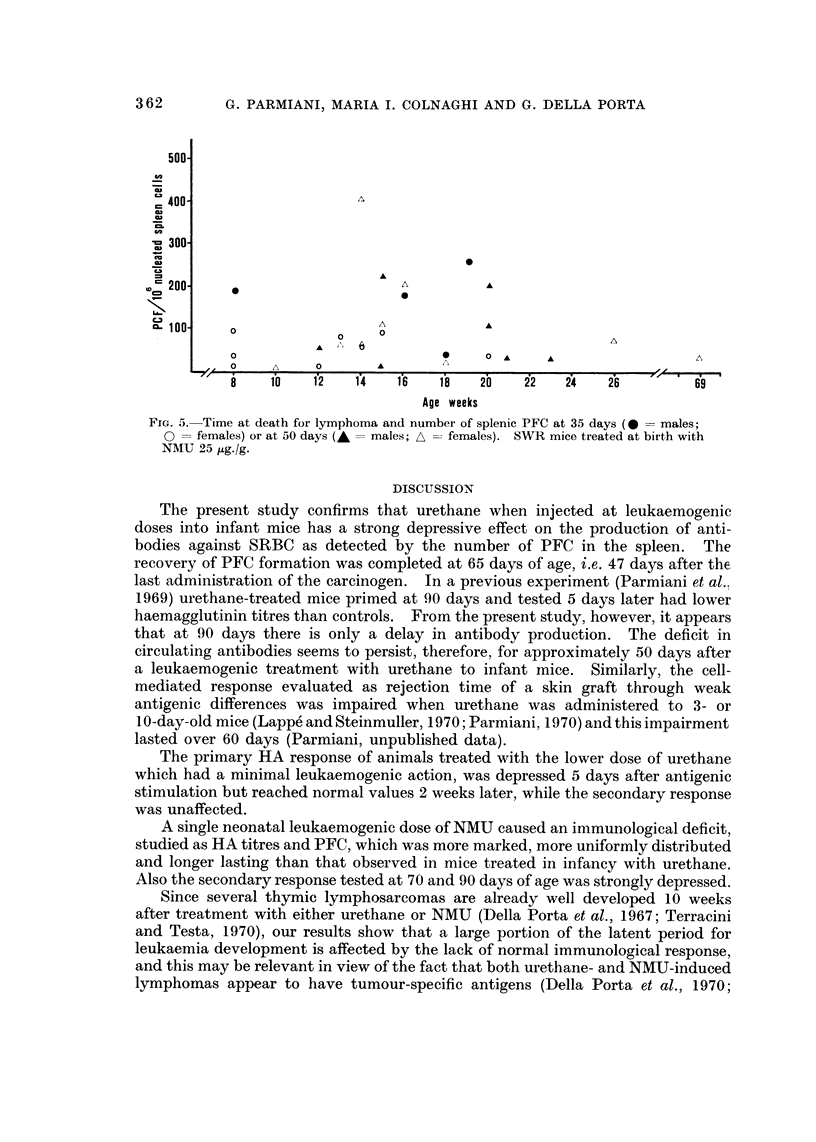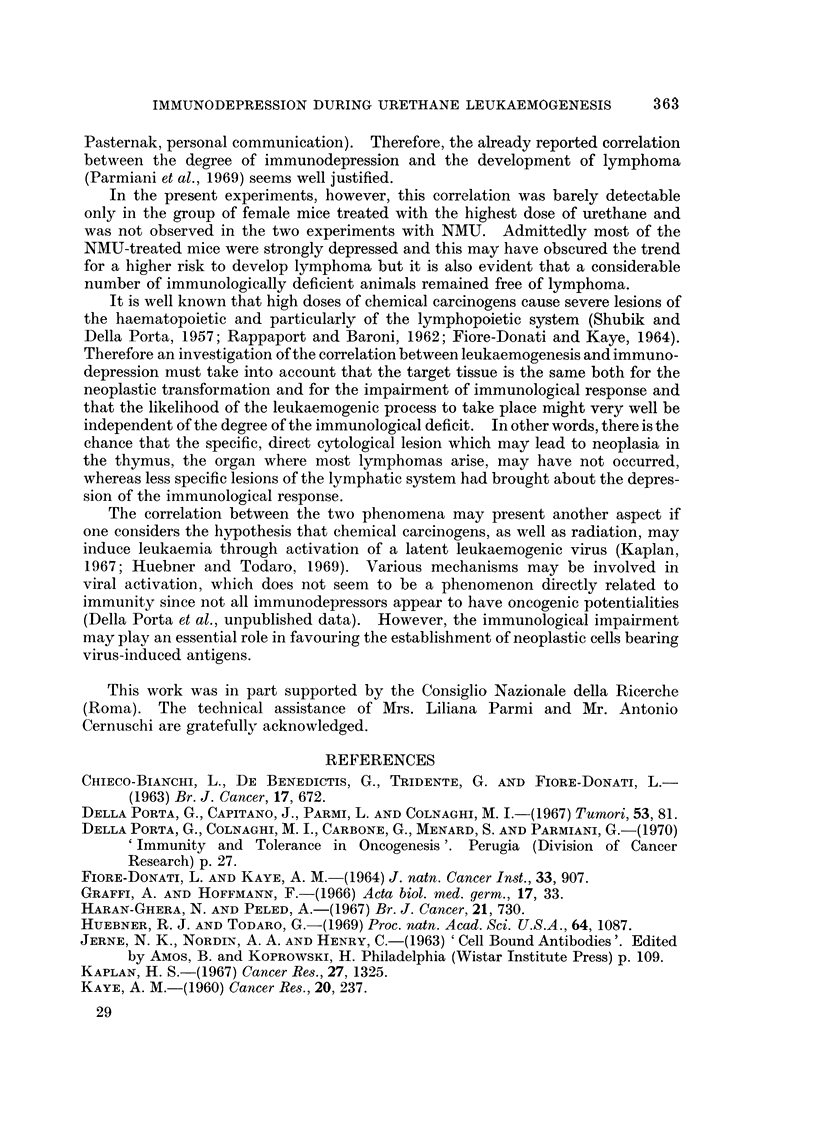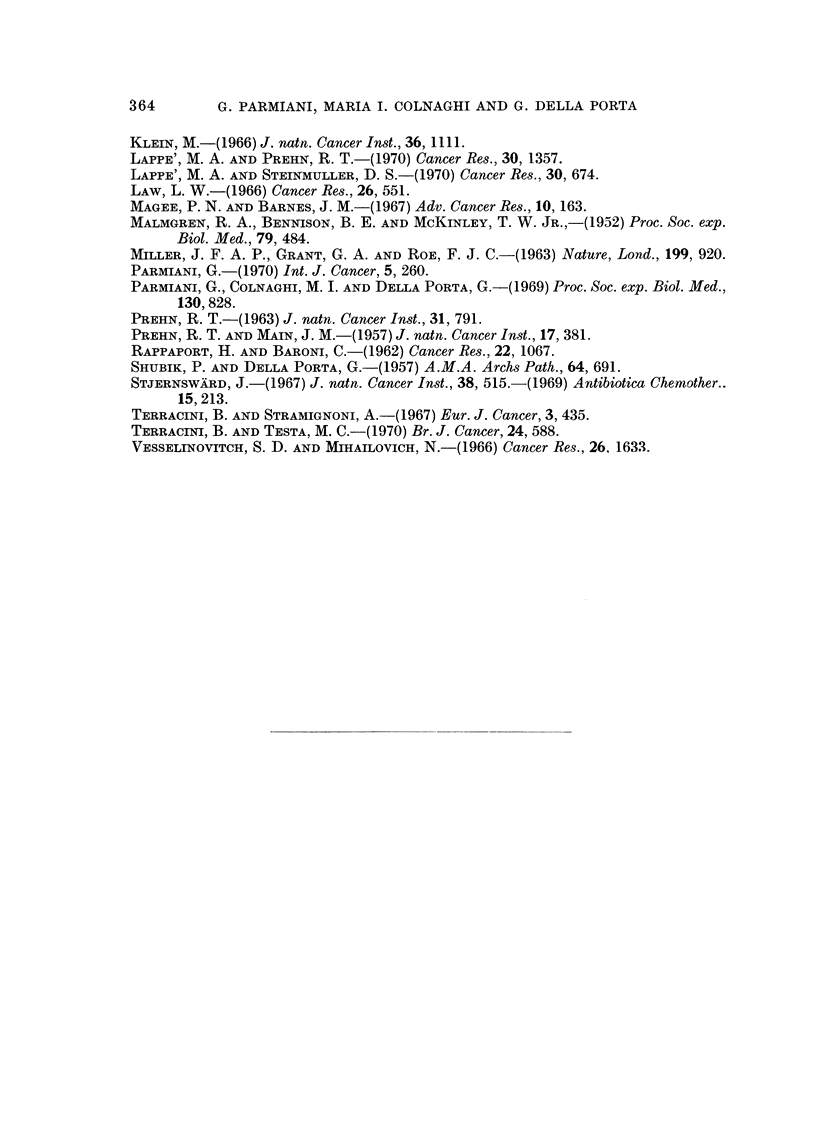# Immunodepression During Urethane and N-Nitrosomethylurea Leukaemogenesis in Mice

**DOI:** 10.1038/bjc.1971.46

**Published:** 1971-06

**Authors:** G. Parmiani, Maria I. Colnaghi, G. Della Porta

## Abstract

Five injections of urethane, 1 mg./g. body weight to suckling mice markedly reduced the primary immune response against sheep red blood cells assessed by splenic plaque forming cells (PFC) determination and haemagglutinin (HA) titration. The immunological impairment lasted for about 50 days after the end of the treatment. The secondary response tested by HA titration was not affected. A lower dose of urethane (0.5 mg./g.) produced only a delay of the primary HA response. A single neonatal dose of N-nitrosomethylurea (NMU) caused a profound immunodepression evaluated as HA titre and number of PFC. Both primary and secondary responses were still depressed when tested at 50 and 90 days of age respectively. No clear correlation between the degree of immunodepression and lymphoma development was found.


### IMMUNODEPRESSION DURING URETHANE AND N-NITROSOMETHYLUREA LEUKAEMOGENESIS IN MICE

G. PARMIANI, MARIA I. COLNAGHI AND G. DELLA PORTA

*From the Section of Experimental Carcinogenesis, Istituto Nazionale per lo Studio e la Cura dei Tumori, 20133 Milano, Italy*

Received for publication January 19, 1971

**SUMMARY.—Five injections of urethane, 1 mg./g. body weight to suckling mice markedly reduced the primary immune response against sheep red blood cells assessed by splenic plaque forming cells (PFC) determination and haemagglutinin (HA) titration. The immunological impairment lasted for about 50 days after the end of the treatment. The secondary response tested by HA titration was not affected. A lower dose of urethane (0·5 mg./g.) produced only a delay of the primary HA response. A single neonatal dose of N-nitrosomethylurea (NMU) caused a profound immunodepression evaluated as HA titre and number of PFC. Both primary and secondary responses were still depressed when tested at 50 and 90 days of age respectively. No clear correlation between the degree of immunodepression and lymphoma development was found.**

THE existence of tumour specific antigens on neoplastic cells is now well established in many experimental systems. Therefore, the immunological apparatus should exert an important role in controlling the development of neoplasia and any interference with immune functions should influence the incidence and latency of tumours as originally proposed by Prehn and Main (1957). This hypothesis has been proved by several studies in mice which included the role of thymectomy on chemical- and virus-induced tumours (Miller *et al.*, 1963; Law, 1966) and the contribution of the immunodepressive effect of carcinogenic substances to their own carcinogenic action (Prehn, 1963; Stjernswärd, 1967). Among the chemical carcinogens, the polycyclic hydrocarbons have been shown to reduce both humoral and cell-mediated immune responses (Stjernswärd, 1969), while other classes of chemical carcinogens have been little investigated from this point of view.

In this report, studies dealing with the effect of two carcinogenic compounds, ethylcarbamate (urethane) and N-nitrosomethylurea (NMU), on the immune response of mice will be presented. Both chemicals are water-soluble and break down rapidly in the animal body (Kaye, 1960; Magee and Barnes, 1967), thereby providing a suitable experimental model to test their action on immunity after their complete elimination from the tissues. Both these compounds have been shown to induce several types of tumours including lymphosarcomas. Whereas NMU can do so after a single neonatal dose (Graffi and Hoffmann, 1966; Terracini and Stramignoni, 1967; Terracini and Testa, 1970), urethane must be administered more than once to suckling mice (Vesselinovitch and Mihailovich, 1966; Della Porta *et al.*, 1967). In fact, when given as a single neonatal dose, urethane elicits only few or no lymphomas but a high incidence of lung adenomas in Swiss mice and hepatomas in C3Hf mice (Chieco-Bianchi *et al.*, 1963; Klein, 1966).

Although the immunodepressive effect of urethane was already demonstrated in 1952 by Malmgren, Bennison and McKinley, only recently urethane carcinogenicity has been studied in relation to its action on the immune system. Previous work from this and other laboratories showed that carcinogenic doses of urethane clearly reduced the immune response either humoral (Haran-Ghera and Peled, 1967; Parmiani *et al.*, 1969) or cell-mediated (Lappé and Steinmuller, 1970; Parmiani, 1970). The depression of humoral and of cell-mediated immunity was also found to be correlated respectively with the development of leukaemia and of lung adenomas (Parmiani *et al.*, 1969; Lappé and Prehn, 1970).

No reports concerning the effects of NMU on the immune functions are available.

In the case of urethane, the aims of the present study were to confirm the immunodepressive effect of urethane on antibody production at cellular level, to verify the duration of the impairment of the primary response and to investigate the relationship between dosage and immunosuppressive action.

In the experiment with NMU, mice were exposed to a single neonatal dose and their immunological status evaluated through the haemagglutinin response against sheep red blood cells and the formation of plaque-forming cells in the spleen.

In both experimental groups an attempt was made to establish a correlation between the degree of immune deficit and lymphoma yield or latency. A similar evaluation for other types of neoplasm was made impossible by the concurrent presence of different kind of tumours in the same animals and by the small number of animals which remained free of tumours.

### MATERIALS AND METHODS

*Animals.*—C3Hf/Dp and SWR/Dp inbred mice of both sexes were used. They were maintained in plastic cages, with tap water and commercial diet in pellets (Mangimi Valle Olona, Castellanza, Italy) *ad libitum.*

*Chemicals.*—Urethane (Merck A. G., Darmstad) was dissolved in distilled sterile water in 6% solution and immediately injected intraperitoneally. The treatment consisted of five injections, at 2 days interval, of 0·5 or 1 mg./g. body weight starting at 10 days of age. Control mice were kept untreated. Recrystalized N-nitrosomethylurea (NMU) was obtained through the courtesy of Mr. P. F. Swann, Courtauld Institute of Biochemistry, Middlesex Hospital Medical School, London. It was dissolved in saline immediately before use at a concentration of 0·1%. Injections were given intraperitoneally at the dose of 25 or 50 $\mu$g./g. body weight, within 24 hours from birth. Control animals received a corresponding volume of saline.

*Immunization procedures.*—Sheep red blood cells (SRBC) were washed three times in saline and injected intraperitoneally as 5% suspension (v/v) in 0·25 ml. of saline.

*Haemagglutinin determination.*—Mice were bled from the retro-orbital plexus. Inactivated sera were diluted in phosphate buffered saline pH 7·2 and serial two-fold dilutions were prepared in Takatsy plastic type plates, the first well contining 0·1 ml. of either 1 : 10 or 1 : 4 diluted antiserum. To each well 0·1 ml. of three times washed 0·5% SRBC was added; haemagglutinin (HA) titres were read after the suspension had settled for 4 hours at room temperature and were controlled after a night.

*Plaque-forming cells determination.*—The number of plaque forming cells (PFC) in the spleen was determined following the technique of Jerne *et al.* (1963). Four days after immunization the spleen was surgically removed under nembutal anaesthesia and weighed. The nucleated cells of each spleen (NSC) were counted and then processed for PFC determination in agar plates. Three plates were prepared from each spleen, and the number of PFC was calculated as a mean of the three plates.

*Pathology.*—Mice were killed when they showed clear symptoms of lymphoma. All survivors were killed at 60–65 weeks of age. Complete autopsy was performed on all animals and specimens were fixed in Bouin, embedded in paraffin and stained with haematoxylin and eosin.

*Statistics.*—Analysis of the results was made by the " t " test. The results were considered statistically significant when $P < 0{\cdot}01$.

### RESULTS

### *Urethane*

*Experiment* 1.—A group of 19 male and 27 female C3Hf mice administered five injections of urethane 1 mg./g. body weight from the 10th to the 18th day of age and a group of 14 male and 13 female untreated controls were immunized with SRBC at 31 days of age. Four additional groups of 6–14 urethane-treated and control mice were immunized at 46, 61, 76 and 91 days of age respectively. The number of PFC was determined 4 days after the immunization.

TABLE I.—*Effect of Five Doses of Urethane Administered During Infancy to C3Hf Mice on Plaque-Forming Spleen Cells*

| Groups | Age at test (days) | No. of mice | PFC/$10^6$ nucleated spleen cells (mean ± S.E.) | No. nucleated cells/spleen × $10^6$ (mean ± S.E.) | Spleen weights (mg.) (mean ± S.E.) | Body weight (g.) (mean ± S.E.) |
|---|---|---|---|---|---|---|
| Controls | 35 | 27 | 476±72 | 89±7 | 123±5 | 17·3±0·3 |
| Urethane | | 46 | 42±5* | 50±5* | 89±6* | 11·1±0·8* |
| Controls | 50 | 14 | 605±68 | 112±8 | 119±5 | 20·0±0·5 |
| Urethane | | 10 | 132±24* | 88±9† | 139±10† | 18·3±1·0† |
| Controls | 65 | 7 | 321±42 | 76±7 | 121±4 | 21·2±0·3 |
| Urethane | | 10 | 395±83† | 66±6† | 122±7† | 20·2±2·3† |
| Controls | 80 | 6 | 386±60 | 92±8 | 125±7 | 22·1±1·2 |
| Urethane | | 10 | 700±107† | 124±4† | 135±4† | 20·4±0·2† |
| Controls | 95 | 14 | 1194±204 | 150±12 | 140±5 | 25·5±0·8 |
| Urethane | | 9 | 727±95† | 125±16† | 102±8† | 21·9±0·9* |

* $P < 0{\cdot}001$.

† Not significant ($P > 0{\cdot}01$).

The results are presented in Table I. At 35 days of age the number of PFC/$10^6$ NSC was ten-fold lower in the urethane-treated mice than in the controls. The immunological impairment was still present at 50 days of age, whereas at 65 days and thereafter the number of PFC did not appear to be significantly diffeient from that of the control groups. Spleen weight, NSC, and body weight of treated animals were markedly reduced in comparison to controls at 35 days only.

The group tested for PFC at 35 days of age, was kept under observation for tumour development for 60 weeks. Six lymphosarcomas appeared among the 13 males and 21 females surviving after splenectomy. As reported in Table II,

TABLE II.—*Relationship Between Development of Thymic Lymphosarcoma and Number of Plaque-Forming Cells at 35 Days in Urethane-treated C3Hf Mice*

| Groups | Sex | Number of mice | PFC/$10^6$ nucleated spleen cells (mean ± S.E.) |
|---|---|---|---|
| Ly mice | ♀ | 4 | 22±6 |
| Non Ly mice | ♀ | 17 | 53±8* |
| Ly mice | ♂ | 2 | (15) (87) |
| Non Ly mice | ♂ | 11 | 48±17 |

Ly mice = Mice which developed lymphoma.
Non Ly mice = Mice which did not develop lymphoma.
*$P < 0{\cdot}01$.

the four female mice which developed lymphoma had been significantly more immunodepressed than those which did not. The occurrence of only two lymphomas among the males prevented any analysis.

*Experiment* 2.—The primary HA response was evaluated in C3Hf mice at various times after the urethane treatment which was terminated at the 18th day of age. The first group of mice was sensitized at 30 days and bled at 35 and 50 days of age; the second one was sensitized at 45 and bled at 50 and 65 days; 3 additional groups were sensitized at 90 days and bled respectively at 95, 100, 110 days of age.

The number of animals in each group and the results are reported in Fig. 1. At 35 days of age, the urethane-treated mice had an immunological deficit of the primary HA response which lasted for the entire 15 days of observation. The same

FIG. 1.—Primary haemagglutinin response to sheep red blood cells in C3Hf mice, either untreated (————) or treated with five injections of urethane 1 mg./g. body weight from the 10th to the 18th day of age (– – – – –). In brackets the number of animals in each experimental group. In this and the following figures, the vertical bars indicate confidence limit at 95%.

behaviour was shown by mice challenged at 45 days of age and tested at 50 and 65 days. On the contrary, when immunized at 90 days of age and tested 5, 10 and 20 days later, the urethane-treated mice showed only a delay in reaching the height of HA titre.

*Experiment* 3.—A group of 25 male and 31 female C3Hf mice were given five doses of urethane 0·5 mg./g. body weight. The mice were then sensitized at 30 and 60 days of age with SRBC and bled at 35, 50, 70 and 90 days.

The haemagglutinin titres are reported in Fig. 2. The data related to control and to 1 mg./g. urethane-treated mice are reported from a previously published experiment (Parmiani, *et al.*, 1969). It appears that whereas the primary response of the animals treated with the higher dose was strongly reduced both at 35 and at 50 days, the lower dose merely produced a delay in the primary response which rose to control values 20 days after antigenic stimulation. The anamnestic response was not impaired as compared with that observed in the control group, whereas mice treated with the higher dose displayed a slight but significant reduction at 70 days only.

Only two lymphomas developed among the animals kept under observation after the immunological tests. This low incidence prevented an analysis of a

FIG. 2.—Primary and secondary haemagglutinin responses to sheep red blood cells in C3Hf mice, either untreated (————) or treated with five injections of urethane, 1 mg./g. body weight (– – – – –) and 0·5 mg./g. body weight (—·—·—·—).

possible correlation between the degree of immunological impairment and the development of leukemia.

*N-nitrosomethylurea*

*Experiment* 4.—Out of 58 newborn C3Hf mice given 50 μg./g. body weight of NMU 28 males and 25 females were weaned and received the antigen at 30 and 60 days of age. HA titre was determined at 35, 50, 70 and 90 days of age. A group of 14 control mice were similarly immunized and tested. As shown in Fig. 3, both the primary and secondary immune responses were strongly depressed in the NMU-treated mice.

Among 26 males and 18 females kept under observation after the test, 26 mice (18 males and 8 females) developed a thymic lymphoma. An attempt to

FIG. 3.—Primary and secondary haemagglutinin responses to sheep red blood cells in C3Hf mice, either untreated (————) or treated with N-nitrosomethylurea 50 μg./g. body weight at birth (– – – – –).

establish a correlation between degree of immunodepression and risk to develop lymphoma was made by using two parameters, the latent period determined as the age at the time of killing for lymphoma, and the tumour incidence. No correlation could be found by plotting the HA titre at 35 days of age against the latency (Fig. 4), since the earliest lymphomas did not occur among the most immunodepressed mice but rather randomly. Lack of convincing evidence for correlation was also found when the HA titre was confronted with the incidence of lymphoma (Table III). Moreover, no significant differences were found between the mean

FIG. 4.—Time at death for lymphoma and primary haemagglutinin response at 35 days of age (● = males; ○ = females), C3Hf mice treated at birth with NMU 50 μg./g.

TABLE III.—*Relationship Between HA Titre at* 35 *Days and Lymphoma Incidence in C*3*Hf Mice, Treated at Birth with N-nitrosomethylurea* 50 *μg./g. Body Weight*

| HA titre ($Log_2$) | Number of mice | Number of mice with lymphoma | % |
|---|---|---|---|
| 2 | 10 | 8 | 80 |
| 3 | 11 | 2 | 18 |
| 4 | 9 | 6 | 66 |
| 5 | 8 | 5 | 63 |
| 6 | 3 | 3 | 100 |
| 7 | 1 | 0 | 0 |
| 8 | — | — | — |
| 9 | 1 | 1 | 100 |
| 11 | 1 | 1 | 100 |

HA titre of mice with lymphoma and those which did not develop lymphoma. In the group with lymphoma the $\log_2$ values of the HA titre were $4 \cdot 2 \pm 1 \cdot 1$ and $3 \cdot 1 \pm 1 \cdot 2$ for males and females while in the group without lymphoma the values were $3 \cdot 7 \pm 1 \cdot 3$ and $3 \cdot 7 \pm 0 \cdot 6$ respectively.

*Experiment* 5.—Newborn SWR mice were given NMU 25 μg./g. body weight or an equivalent volume of saline. At weaning the NMU-treated mice were divided into two groups. The first group of 11 males and 15 females received SRBC when 31 days old; the second one, containing 16 males and 21 females was immunized at 46 days. Twelve control mice for the first group and 19 for the second one were immunized in the same manner. Four days later the mice underwent splenectomy for PFC determination. As can be seen from Table IV, there was a striking reduction of the number of PFC in both NMU-treated groups. Also NSC were clearly reduced at 35 and 50 days, whereas the reduction of spleen weight was significant at 50 days only.

TABLE IV.—*Effects of N-nitrosomethylurea 25 μg./g. Body Weight Administered at Birth on Plaque-Forming Spleen Cells of SWR Mice*

| Groups | Age at test (days) | Number of mice | PFC/$10^6$ nucleated spleen cells (mean ± S.E.) | No. of nucleated cells/spleen × $10^6$ (mean ± S.E.) | Spleen weight (mg.) (mean ± S.E.) | Body weight (g.) (mean ± S.E.) |
|---|---|---|---|---|---|---|
| Controls | 35 | 12 | 673±63 | 137±18 | 99±5 | 13·8±0·4 |
| NMU | | 26 | 83±13* | 65±9* | 83±7‡ | 10·0±0·4* |
| Controls | 50 | 19 | 672±54 | 193±17 | 145±9 | 17·3±0·2 |
| NMU | | 37 | 162±19* | 125±9* | 106±6† | 16·1±0·5‡ |

* $P < 0 \cdot 001$.

† $P < 0 \cdot 005$.

‡ Not significant ($P > 0 \cdot 01$).

TABLE V.—*Relationship Between Development of Thymic Lymphosarcoma and Number of Plaque-Forming Cells in SWR Mice Treated at Birth with N-nitrosomethylurea 25 μg./g. Body Weight.*

| Experimental groups | Number of mice | Age at test (days) | PFC/$10^6$ N.S.C. (mean ± S.E.) |
|---|---|---|---|
| Ly mice | 12 | 35 | 90±23 |
| Non Ly mice | 5 | 35 | 120±44* |
| Ly mice | 16 | 50 | 100±27 |
| Non Ly mice | 21 | 50 | 143±19* |

Ly mice = Mice which developed lymphomas.
Non ly Mice = Mice which did not develop lymphomas.
* Not significant ($P > 0 \cdot 01$).

As reported in Table V, the average number of PFC/$10^6$ NSC at 35 days was not significantly different between mice which eventually developed lymphomas and those which did not. Within the limits of the high immunodepression observed in all treated animals, a random distribution was obtained when the number of PFC/$10^6$ NSC at 35 and 50 days was plotted against the time of lymphoma appearance (Fig. 5).

FIG. 5.—Time at death for lymphoma and number of splenic PFC at 35 days (● = males; ○ = females) or at 50 days (▲ = males; △ = females). SWR mice treated at birth with NMU 25 μg./g.

### DISCUSSION

The present study confirms that urethane when injected at leukaemogenic doses into infant mice has a strong depressive effect on the production of antibodies against SRBC as detected by the number of PFC in the spleen. The recovery of PFC formation was completed at 65 days of age, *i.e.* 47 days after the last administration of the carcinogen. In a previous experiment (Parmiani *et al.*, 1969) urethane-treated mice primed at 90 days and tested 5 days later had lower haemagglutinin titres than controls. From the present study, however, it appears that at 90 days there is only a delay in antibody production. The deficit in circulating antibodies seems to persist, therefore, for approximately 50 days after a leukaemogenic treatment with urethane to infant mice. Similarly, the cell-mediated response evaluated as rejection time of a skin graft through weak antigenic differences was impaired when urethane was administered to 3- or 10-day-old mice (Lappé and Steinmuller, 1970; Parmiani, 1970) and this impairment lasted over 60 days (Parmiani, unpublished data).

The primary HA response of animals treated with the lower dose of urethane which had a minimal leukaemogenic action, was depressed 5 days after antigenic stimulation but reached normal values 2 weeks later, while the secondary response was unaffected.

A single neonatal leukaemogenic dose of NMU caused an immunological deficit, studied as HA titres and PFC, which was more marked, more uniformly distributed and longer lasting than that observed in mice treated in infancy with urethane. Also the secondary response tested at 70 and 90 days of age was strongly depressed.

Since several thymic lymphosarcomas are already well developed 10 weeks after treatment with either urethane or NMU (Della Porta *et al.*, 1967; Terracini and Testa, 1970), our results show that a large portion of the latent period for leukaemia development is affected by the lack of normal immunological response, and this may be relevant in view of the fact that both urethane- and NMU-induced lymphomas appear to have tumour-specific antigens (Della Porta *et al.*, 1970;

Pasternak, personal communication). Therefore, the already reported correlation between the degree of immunodepression and the development of lymphoma (Parmiani *et al.*, 1969) seems well justified.

In the present experiments, however, this correlation was barely detectable only in the group of female mice treated with the highest dose of urethane and was not observed in the two experiments with NMU. Admittedly most of the NMU-treated mice were strongly depressed and this may have obscured the trend for a higher risk to develop lymphoma but it is also evident that a considerable number of immunologically deficient animals remained free of lymphoma.

It is well known that high doses of chemical carcinogens cause severe lesions of the haematopoietic and particularly of the lymphopoietic system (Shubik and Della Porta, 1957; Rappaport and Baroni, 1962; Fiore-Donati and Kaye, 1964). Therefore an investigation of the correlation between leukaemogenesis and immunodepression must take into account that the target tissue is the same both for the neoplastic transformation and for the impairment of immunological response and that the likelihood of the leukaemogenic process to take place might very well be independent of the degree of the immunological deficit. In other words, there is the chance that the specific, direct cytological lesion which may lead to neoplasia in the thymus, the organ where most lymphomas arise, may have not occurred, whereas less specific lesions of the lymphatic system had brought about the depression of the immunological response.

The correlation between the two phenomena may present another aspect if one considers the hypothesis that chemical carcinogens, as well as radiation, may induce leukaemia through activation of a latent leukaemogenic virus (Kaplan, 1967; Huebner and Todaro, 1969). Various mechanisms may be involved in viral activation, which does not seem to be a phenomenon directly related to immunity since not all immunodepressors appear to have oncogenic potentialities (Della Porta *et al.*, unpublished data). However, the immunological impairment may play an essential role in favouring the establishment of neoplastic cells bearing virus-induced antigens.

This work was in part supported by the Consiglio Nazionale della Ricerche (Roma). The technical assistance of Mrs. Liliana Parmi and Mr. Antonio Cernuschi are gratefully acknowledged.

### REFERENCES

Chieco-Bianchi, L., De Benedictis, G., Tridente, G. and Fiore-Donati, L.—(1963) *Br. J. Cancer*, **17**, 672.

Della Porta, G., Capitano, J., Parmi, L. and Colnaghi, M. I.—(1967) *Tumori*, **53**, 81.

Della Porta, G., Colnaghi, M. I., Carbone, G., Menard, S. and Parmiani, G.—(1970) 'Immunity and Tolerance in Oncogenesis'. Perugia (Division of Cancer Research) p. 27.

Fiore-Donati, L. and Kaye, A. M.—(1964) *J. natn. Cancer Inst.*, **33**, 907.

Graffi, A. and Hoffmann, F.—(1966) *Acta biol. med. germ.*, **17**, 33.

Haran-Ghera, N. and Peled, A.—(1967) *Br. J. Cancer*, **21**, 730.

Huebner, R. J. and Todaro, G.—(1969) *Proc. natn. Acad. Sci. U.S.A.*, **64**, 1087.

Jerne, N. K., Nordin, A. A. and Henry, C.—(1963) 'Cell Bound Antibodies'. Edited by Amos, B. and Koprowski, H. Philadelphia (Wistar Institute Press) p. 109.

Kaplan, H. S.—(1967) *Cancer Res.*, **27**, 1325.

Kaye, A. M.—(1960) *Cancer Res.*, **20**, 237.

KLEIN, M.—(1966) *J. natn. Cancer Inst.*, **36**, 1111.
LAPPE', M. A. AND PREHN, R. T.—(1970) *Cancer Res.*, **30**, 1357.
LAPPE', M. A. AND STEINMULLER, D. S.—(1970) *Cancer Res.*, **30**, 674.
LAW, L. W.—(1966) *Cancer Res.*, **26**, 551.
MAGEE, P. N. AND BARNES, J. M.—(1967) *Adv. Cancer Res.*, **10**, 163.
MALMGREN, R. A., BENNISON, B. E. AND MCKINLEY, T. W. JR.,—(1952) *Proc. Soc. exp. Biol. Med.*, **79**, 484.
MILLER, J. F. A. P., GRANT, G. A. AND ROE, F. J. C.—(1963) *Nature, Lond.*, **199**, 920.
PARMIANI, G.—(1970) *Int. J. Cancer*, **5**, 260.
PARMIANI, G., COLNAGHI, M. I. AND DELLA PORTA, G.—(1969) *Proc. Soc. exp. Biol. Med.*, **130**, 828.
PREHN, R. T.—(1963) *J. natn. Cancer Inst.*, **31**, 791.
PREHN, R. T. AND MAIN, J. M.—(1957) *J. natn. Cancer Inst.*, **17**, 381.
RAPPAPORT, H. AND BARONI, C.—(1962) *Cancer Res.*, **22**, 1067.
SHUBIK, P. AND DELLA PORTA, G.—(1957) *A.M.A. Archs Path.*, **64**, 691.
STJERNSWÄRD, J.—(1967) *J. natn. Cancer Inst.*, **38**, 515.—(1969) *Antibiotica Chemother.*. **15**, 213.
TERRACINI, B. AND STRAMIGNONI, A.—(1967) *Eur. J. Cancer*, **3**, 435.
TERRACINI, B. AND TESTA, M. C.—(1970) *Br. J. Cancer*, **24**, 588.
VESSELINOVITCH, S. D. AND MIHAILOVICH, N.—(1966) *Cancer Res.*, **26**, 1633.